# Effects of repetitive practice of motor tasks on somatosensory gating

**DOI:** 10.3389/fnhum.2023.1131986

**Published:** 2023-03-29

**Authors:** Mayu Akaiwa, Yuya Matsuda, Hidekazu Saito, Eriko Shibata, Takeshi Sasaki, Kazuhiro Sugawara

**Affiliations:** ^1^Graduate School of Health Sciences, Sapporo Medical University, Sapporo, Japan; ^2^Department of Occupational Therapy, School of Health Science, Sapporo Medical University, Sapporo, Japan; ^3^Department of Physical Therapy, Faculty of Human Science, Hokkaido Bunkyo University, Eniwa, Japan; ^4^Department of Physical Therapy, School of Health Science, Sapporo Medical University, Sapporo, Japan

**Keywords:** somatosensory evoked potentials (SEPs), gating, electroencephalogram (EEG), motor learning, motor performance

## Abstract

**Introduction:**

During voluntary muscle contraction, the amplitude of the somatosensory evoked potential (SEP) is reduced by inhibiting sensory information from a peripheral nerve supplying the contracted muscle. This phenomenon is called “gating.” We reported that participants with good motor skills indicated strong suppression of somatosensory information. The present study investigated the effects of motor performance improvement following repetitive practice on the SEP amplitude.

**Methods:**

The ball rotation task (BR task) was practiced by 15 healthy participants repetitively. SEPs were recorded before (pre) and after (post) repetitive practice.

**Results:**

The BR task performance was significantly improved and the required muscle activation to perform the task was significantly reduced after the repetitive practice. The degree of gating was not significant between pre and post- for the SEP amplitude. A significant correlation was found between changes in SEP amplitude from pre to post and performance improvement.

**Discussion:**

After repetitive practice, the degree of gating did not change, but the performance of the BR task improved, and the muscle activity required for the BR task decreased. These results suggest that repetitive practice does not change the degree of gating but changes the mechanism of gating. Furthermore, they indicate that suppression of the somatosensory area may play a role in improving task performance.

## Introduction

Somatosensory information is necessary for motor control (Rothwell et al., 1982; Johansson and Flanagan, 2009). The motor system regulates sensory inflow from the periphery to the somatosensory cortex during movement (Papakostopoulos et al., 1975; Rushton et al., 1981). Previous studies have reported that somatosensory evoked potentials (SEPs) and somatosensory evoked fields are attenuated during voluntary movements compared to the resting state (Papakostopoulos et al., 1975; Rushton et al., 1981). This phenomenon is called “gating.” The gating in voluntary movements has been reported to be caused by two factors. Centrifugal gating occurs through a central mechanism in which output from motor cortical regions suppresses afferent input from a peripheral nerve (Jones et al., 1989). The second is centripetal gating, which is due to competition between the input from the presented stimulus and the afferent proprioceptive feedback from the movement itself (Jones et al., 1989). A previous study reported that gating was changed dependent on the strength of the muscular contraction (Sugawara et al., 2016). Additionally, this study suggested that this gating is generated by both gating mechanisms, the centrifugal gating and the centripetal gating.

We demonstrated that participants with better dexterous movement skills indicated the stronger suppression of somatosensory information (Akaiwa et al., 2022b). Our findings suggest a relationship between the gating and the activity of sensorimotor area in the motor learning stage. A recent study revealed an increased a excitability of primary somatosensory cortex (S1) early in learning and increased primary motor cortex (M1) excitability later while achieving a high degree of precision in learning (Ohashi et al., 2019). Furthermore, a previous study indicated that premotor area (PMA) and supplementary motor area (SMA) excitability changes during the motor skill learning process (Grafton et al., 1992). The dexterous finger movement skills were improved when PMA or SMA was stimulated using non-invasive brain stimulation (Pavlova et al., 2014; Hupfeld et al., 2017). Thus, changes in the motor-related cortical area may lead to altering the suppression of somatosensory information during the motor skill learning process. The present study focused on changes in gating following repetitive practice of motor tasks. We investigated the relationship between the suppression of somatosensory information and motor skill acquisition using SEPs. In previous studies, SEP components of P25 (P22 in Frontal) reflecting activity in area 3b, N30 in frontal reflecting activity in SMA and PMA, N33 in parietal reflecting activity in area 1, and P45 (P40 in Frontal) reflecting activity in the posterior parietal cortex were reported to be gated during voluntary movement (Nishihira et al., 1997; Kida et al., 2006). Since these regions are thought to be involved in somatosensory information processing during voluntary movements, we hypothesized that gating would be induced in P22, N30, P40 in F3 and P25, N33, P45 in C3′ and P3 during the BR task in this study. Furthermore, previous studies have shown that the P45 component at the C3′ and P3 electrodes is significantly reduced in subjects with good motor skills compared with those who perform poorly (Akaiwa et al., 2022b). In the present study, we also hypothesized that gating in P45 at C3′ and P3 would be stronger (i.e., the amplitude would decrease) after repetitive practice.

## Materials and methods

### Participants

The minimum sample size for the present study was determined using G*power software from partial η-squared that was determined by the previous study (Tennant et al., 2021). The effect size was fixed to 0.35. This analysis revealed a minimum sample size of 15. We recruited 15 healthy young adults [age (mean ± standard deviation): 22.6 ± 2.2 years; 8 males, 7 females], all of whom provided written informed consent. The study was approved by the Ethics Committee of Sapporo Medical University (No. 2-1-91) and with the 1964 Helsinki declaration and its later amendments or comparable ethical standards.

### Stimulation

Electrical stimuli were applied to the right median nerve at the right wrist for a duration of 0.2 ms. Stimulus intensity was adjusted to a motor threshold (Wasaka et al., 2017). The median nerve stimulation intensity was 3.75 ± 0.67 mA (Mean ± SD). Stimuli were randomly presented at 1–3 Hz. The participants were asked to concentrate on the dexterity movement and were instructed not to pay any attention to the electrical stimuli.

### Task

They performed the ball rotation task (BR task) using the right fingers. The BR task involved rotating two wooden balls counterclockwise in the palm of the right hand as quickly as possible (Kawashima et al., 1998; Naito et al., 2020). The duration of each set was 1 min. The participants performed 12 sets of BR tasks. Stimulus intensity and location were checked before and after recording throughout the experiment to ensure that there was no stimulus displacement. A video of the participants rotating the two balls was recorded, and the number of ball rotations was counted. Then, we removed the set, and instructed the participants to start over again if they dropped the ball. Due to the possibility of shifting the stimulation site, the participant was instructed not to pronate or supinate the forearm or flex or extend the wrist from a starting position during the BR task.

### Experimental procedure

**FIGURE 1:**
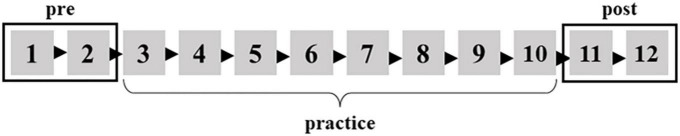
The BR task was carried out 12 sets in total. The mean of the first two sets is defined as pre. The mean of the last two sets is defined as post.

Participants were seated in a reclining chair. The stimulator was placed on the ventral side of the wrist joint and fixed with Velcro. Participants rested with their forearms on a table placed to their right. The first part of the experiment recorded resting SEPs for 2 min (rest). Then, SEPs were recorded at a set of 1 and 2 during the BR task (pre) (Figure 1). Also, SEPs were recorded at sets of 11 and 12 during the BR task (post) after the participants practiced the BR task for 8 sets (Figure 1). The participants were stimulated at approximately 120 per set. The stimulation was delivered 240 times in pre or post.

### Recording and analysis

The Neuropack system was used for all electrophysiological recordings (Nihon Kohden, Tokyo, Japan). The electroencephalography (EEG) was measured using Ag/AgCl electrodes placed over three scalp sites, F3, C3′ (2 cm posterior to C3), and P3. A reference electrode was placed at the left earlobe (A1). The electrode impedance was kept below 5 kΩ at all recording sites. The electrooculogram (EOG) was recorded from the right suborbital region. Trials with EOG waveform exceeding 80 μV were rejected (Akaiwa et al., 2022a). EEG signals were recorded with a 0.1–200 Hz band-pass filter at a sampling rate of 1,000 Hz. The SEP analysis window was from 50 ms before to 100 ms after the stimulus onset. The amplitude of four SEP components were calculated by peak-to-peak methods (F3: N18, P22, N30, and P45; C3’, P3: N20, P25, N33, and P45). Also, the amplitude of each component was measured from the preceding peaks (Kirimoto et al., 2014; Lei et al., 2018).

Electromyogram (EMG) (DL-140; 4assist, Japan) was recorded using a pair of Ag/AgCl electrodes (Blue-sensor NF-00; Ambu, Denmark) placed over the right abductor pollicis brevis muscle (APB) because muscle activity affects gating. Furthermore, EMG over the right flexor carpi radialis (FCR) was recorded to detect the joint over-motion of the right wrist and forearm. EMG signals were digitized and recorded using PowerLab and LabChart software (ADInstruments, New Zealand). Maximal voluntary contraction (MVC) of > 5 s of each muscle was recorded at first. EMG signals were sampled at 1,000 Hz and band-pass filtered at 10–300 Hz. Full wave was rectified. Then, EMG signals were normalized to MVC. The averaged normalized EMG was calculated for each set. We defined the average of 1 and 2 sets as pre and 11 and 12 sets as post. The effect of task performance was examined on repetitive practice. The average number of ball rotations in sets 1 and 2 were defined as pre- and sets 11 and 12 as the post.

Statistical analyses were performed using the IBM Statistical Package for the Social Sciences version 25 software (IBM Corp., New York, NY, USA). To assess normality, the Shapiro–Wilk test was used. Next, Mauchly’s test of sphericity was used to analyze the assumption of sphericity prior to repeated measures analysis of variance (ANOVA). The Greenhouse–Geisser fit was used to correct for this violation, as significant test results indicated that the sphericity assumption had been violated. Then, one-way repeated measure ANOVAs were performed to determine the effect of condition (rest, pre, and post) on the SEP component amplitudes. For *post hoc* comparisons, paired *t*-tests were used with Bonferroni adjustment for multiple comparisons. Also, to examine the effect of EMG or the number of ball rotations on the repetitive practice, paired-*t* tests were performed on the EMG and the number of ball rotations. A previous study showed a correlation between the performance of BR tasks and the degree of P45 gating at C3’and P3 electrodes (Akaiwa et al., 2022b). Therefore, in this study, we performed a correlation analysis between the changes in P45 amplitude at C3’and P3 electrodes and the number of ball rotations to investigate how repetitive practice affects P45. Moreover, we performed a correlation analysis of the changes in P45 at C3′ and the changes in the muscle activity of APB. Statistical significance was set at *P*-values of < 0.05.

## Results

### Behavioral data

**FIGURE 2:**
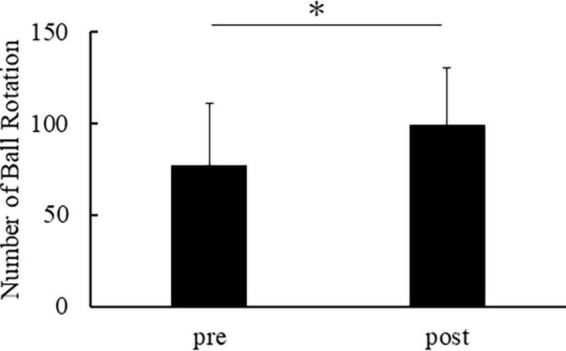
Number of ball rotations in pre and post. The horizontal axis indicates the number of ball rotations. The error bar indicates the standard deviation. Asterisks indicate significant differences (*p* < 0.05).

Figure 2 shows the number of ball rotations of all participants. All participants demonstrated an enhancement in the number of ball rotations after repetitive practice. The number of ball rotations in the post was significantly increased compared to the pre [*t*(14) = −8.373, *p* < 0.001].

### EMG

**FIGURE 3:**
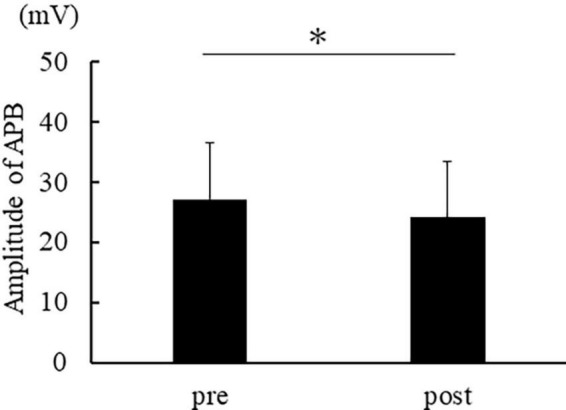
The average of APB muscle activation. The error bar indicates the standard deviation. Asterisks indicate significant differences (*p* < 0.05).

**FIGURE 4:**
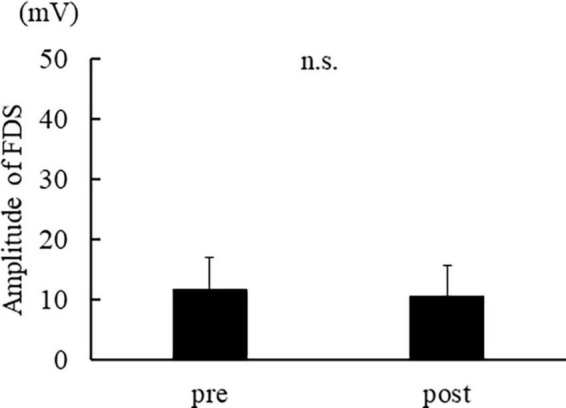
The average of FDS muscle activation. The error bar indicates the standard deviation.

Figure 3 shows the muscle activity in APB, and Figure 4 shows the activity in FDS. The muscle activity in the post-was significantly decreased compared to the pre- in APB [*t*(14) = 2.974, *p* = 0.010]. No significant difference was found in FDS [*t*(14) = 1.783, *p* = 0.096].

### SEPs

**FIGURE 5:**
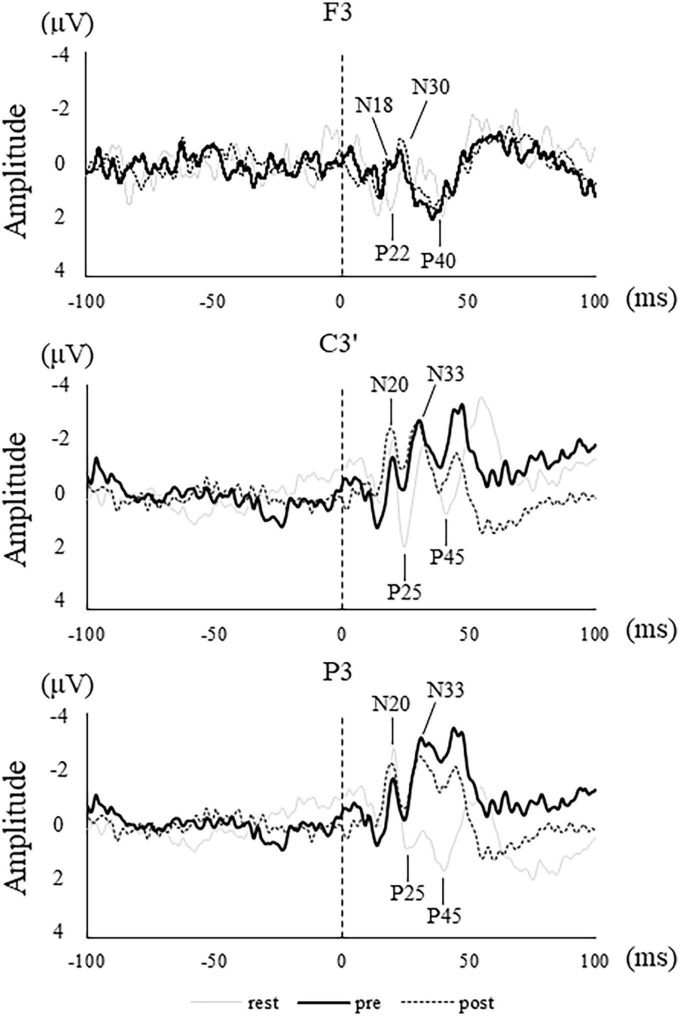
SEPs waveform in a representative participant in rest (thin solid line), pre (solid line), and post (dotted line) at each electrode site. Vertical dotted lines indicate stimulus onset.

**FIGURE 6:**
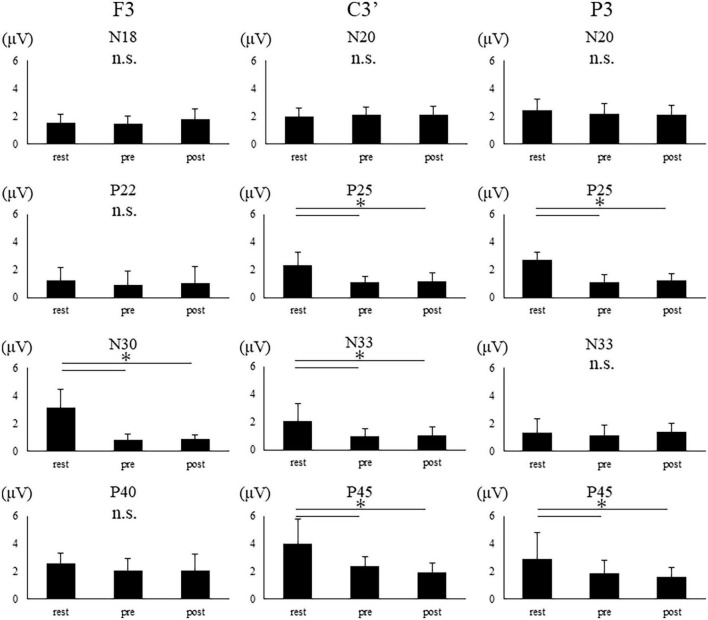
SEP amplitudes of the two groups in each condition at each electrode site. The error bar indicates standard deviations. Asterisks indicate significant differences (*p* < 0.05).

Figure 5 shows the SEP waveform in a representative participant. Figure 6 shows the average and standard deviation of SEP amplitude for each component. Four participants were rejected for F3 electrode analysis due to artifacts. Furthermore, due to an undetectable peak, one participant was rejected for P25 and N33 analysis at the C3 electrode.

The amplitudes of N18 at F3 and N20 at C3′ and P3 demonstrate no main effect [F3, *F*(2,20) = 2.718, *p* = 0.090; C3’, *F*(2,28) = 0.794, *p* = 0.462; P3, *F*(1.459,20.430) = 3.339, *p* = 0.069]. The amplitude of N30 showed main effect at F3 electrode [*F*(1.149,10.342) = 40.270, *p* < 0.001]. The *post-hoc* test revealed a significant reduction in the N30 amplitude in pre and post compared to rest (rest vs. pre, *p* < 0.001; rest vs. post, *p* < 0.001). Further, N22, and P40 at F3 exhibit no main effect. The amplitudes of P25, N33, and P45 revealed a main effect at C3′ electrode [P25, *F*(2,26) = 20.809, *p* < 0.001; N33, *F*(1.271,76.518) = 8.526, *p* = 0.001; P45, *F*(1.308,18.308) = 15.026, *p* < 0.001]. The *post-hoc* test revealed that each amplitude was significantly reduced in pre and post compared to rest (P25: rest vs. pre, *p* < 0.001; rest vs. post, *p* < 0.001; N33: rest vs. pre, *p* = 0.002; rest vs. post, *p* = 0.003; P45: rest vs. pre, *p* = 0.001; rest vs. post, *p* < 0.001). The amplitudes of P25 and P45 revealed a main effect at P3 electrode [P25, *F*(2,28) = 58.670, *p* < 0.001; P45, *F*(2,28) = 6.609, *p* = 0.004]. The *post-hoc* test revealed that P25 and P45 amplitudes were significant reductions in pre- and post-compared to rest (P25: rest vs. pre, *p* < 0.001; rest vs. post, *p* < 0.001; P45: rest vs. pre, *p* = 0.035; rest vs. post, *p* = 0.005). No significant differences were found between pre and post at all electrodes.

**FIGURE 7:**
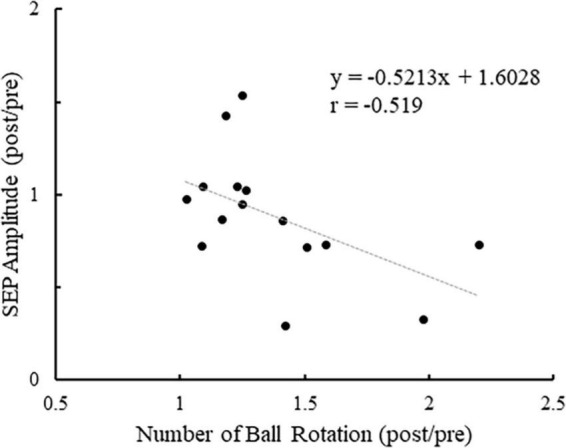
The plot of the change in the number of ball rotations relative to the change in P45 amplitude at the C3′ electrode for all participants. Values of > 1 indicate enhancement from pre to post.

**FIGURE 8:**
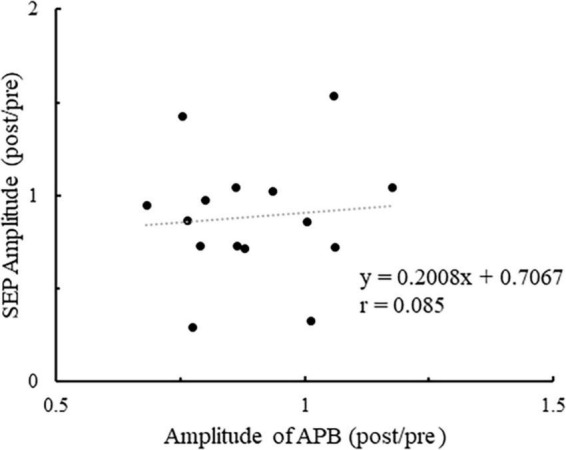
The plot of the change in the muscle activity of APB relative to the change in P45 amplitude at the C3′ electrode for all participants. Values of > 1 indicate enhancement from pre- to post.

Figure 7 shows the plot of the change in the number of ball rotations relative to the change in P45 amplitude at C3′ for all participants. Also, Pearson’s correlation analyses revealed a significant negative correlation between the number of ball rotations and the P45 at C3′ (*p* = 0.047. *r* = −0.519). Figure 8 shows the plot of the changes in P45 at C3′ and the changes in muscle activity of APB for all participants. There was no significant correlation between the changes in muscle activity and the changes in P45 at C3′ (*p* = 0.764, *r* = 0.085).

## Discussion

The present study examined the relationship between task performance and gating throughout repetitive practice and revealed the following: (1) The number of ball rotations in post-was significantly increased compared to pre, and APB muscle activity in post-was significantly decreased compared to pre. (2) N30 at F3 electrode; P25, N33, and P45 at C3′ electrode; and P25 and P45 at P3 electrode during the BR task were attenuated compared to rest. (3) No significant difference was found between pre and post in SEP components. (4) P45 amplitude at C3′ electrode showed a significantly negative correlation to the enhancement of the BR task performance.

A previous study revealed that the participants with better dexterous movement skills displayed stronger suppression of somatosensory information (Akaiwa et al., 2022b). Therefore, we hypothesized that repetitive practice would improve task performance and increase the degree of gating. However, our results in the present study revealed no significant difference in the degree of gating before and after repetitive practice despite enhanced performance in the BR task. SEP amplitudes attenuation could be caused by two mechanisms: centripetal and centrifugal gating (Jones et al., 1989). After repetitive practice, centripetal gating would be more contributive due to greater input *via* Ia afferent fibers induced by finger movement and from the skin induced by the balls contacting the palm.

In contrast, due to APB activity reduction, centrifugal gating from M1 would exhibit less contribution. Several previous studies revealed that PMA and SMA excitability could be altered after repetitive practice using manual tasks (Grafton et al., 1992; Jenkins et al., 1994). Previous studies indicated that PMA exhibited greater activation when subjects learned new sequences than during the pre-learned task performance (Jenkins et al., 1994). PMA makes a greater contribution when performance is guided by external cues, which are the earliest stages of motor learning. By contrast, a previous study reported greater activation in the SMA during an automatic performance than the earliest stages of learning (Jenkins et al., 1994). SMA makes a greater contribution when movements become smooth, coordinated, and often more rapid, which is the overlearned or automatic phase. Accordingly, it is possible that PMA and SMA in this study showed greater activation in pre and post, respectively. Thus, our findings suggest that PMA compared to SMA may contribute to centrifugal gating in pre and the relationship may be reversed in post. Therefore, our results suggest that repetitive practice does not change the degree of gating, but it changes the mechanism of gating. However, this suggestion is limited because it does not record the activation of movement-related cortical areas.

The recordings at C3′ and P3 electrodes suggested that SEP components of P25, N33, and P45 reflect activated 3b, 1, and posterior parietal cortex (PPC) (Jones et al., 1978; Mauguière et al., 1983; Allison et al., 1991). Next, some studies reported no change in the amplitude of P25 (which corresponds to M20 in the MEG) (Kakigi et al., 2000; Sugawara et al., 2016), while others described an attenuation of the amplitude (Seyal et al., 1987; Hoshiyama and Sheean, 1998; Wasaka et al., 2017). In contrast, Attenuation of N33 and P45 amplitudes by voluntary movements has been consistently reported (Kirimoto et al., 2014; Sugawara et al., 2016; Lei and Perez, 2017; Akaiwa et al., 2022b). Our findings suggest that somatosensory processing areas are regulated during the BR tasks, regardless of task skill.

Accordingly, the present study revealed that N30 amplitude at F3 during the BR task was attenuated compared to rest. A previous study indicates that the SMA could play an important role in the frontal N30 generation (Chéron and Borenstein, 1992; Legon et al., 2013). Also, N30 amplitude was reported to be attenuated during mental movement simulation, in which regional blood flow in SMA increases (Chéron and Borenstein, 1992). Furthermore, a previous study supports that PMA also is involved in frontal N30 generation (Urushihara et al., 2006). Reportedly, 0.2 Hz repetitive transcranial magnetic stimulation over the PMA increased N30 amplitude (Urushihara et al., 2006). Therefore, we suggest that activation of PMA, and/or SMA changes during the BR task following repetitive practice.

Our results revealed a significant negative correlation between the change in the number of ball rotations and P45 amplitude at C3′ electrode. A previous study suggests that P45 may reflect PPC activation (Inui et al., 2004). Recent studies revealed that anodal transcranial direct current stimulation (tDCS) over PPC can impair task performance (Grasso et al., 2020; Grasso et al., 2021). The facilitation of PPC activation by anodal tDCS was suggested to negatively affect task performance. Therefore, the activity of PPC during task performance is thought to influence performance. In our results, participants with better task performance after repetitive practice were more suppressed PPC during the BR task. This study suggests that suppression of PPC is important during tasks in improving task performance.

This study exhibits several limitations. One of the limitations of this study is that recordings were not performed using multi-channel electrodes. Multi-channel recording allows source localization. In addition, the source of noise can be removed so that data can be recorded with improved noise and signal separation. In a future study, we will use a multi-channel electroencephalograph to measure and analyze the data more accurately. Secondly, Participants were instructed to avoid forearm and wrist movements as much as possible to avoid misalignment of the right median nerve stimulation, and we confirmed that the intensity of stimulation did not change before and after the BR task. However, the possibility that slight movements of the wrist joint could move the placement of the stimulation electrodes and thus modify the arrangement of the generated electric field is a limitation of this study. Next, we could not possibly distinguish which gating effect contributed more strongly between centripetal or centrifugal gating because each mechanism would change following repetitive practice of the BR task. Hence, in a future study, we should change the motor task. Moreover, Activity in movement-related cortical areas was not recorded. We could not detect activity in movement-related cortical areas because we only assessed SEP-related sensory input. Therefore, future studies should focus on such investigations using instruments with high spatial resolution and revealing the relationship among motor skill, the activity of movement-related cortical area, and somatosensory gating.

## Conclusion

This study investigated the effects of motor performance improvement following repetitive practice on the SEP amplitude. After repetitive practice, the degree of gating did not change, but the performance of the BR task improved, and the muscle activity required for the BR task decreased. These results suggest that repetitive practice does not change the degree of gating but changes the mechanism of gating. Furthermore, our correlation results indicate that the suppression of somatosensory area is important for improving task performance.

## Data availability statement

The original contributions presented in this study are included in the article/Supplementary material, further inquiries can be directed to the corresponding author.

## Ethics statement

This study was approved by the Ethics Committee of Sapporo Medical University (No. 2-1-91) and with the 1964 Helsinki Declaration and its later amendments or comparable ethical standards. The patients/participants provided their written informed consent to participate in this study.

## Author contributions

MA, KS, YM, and HS: material preparation and data collection. MA: formal analysis and the first draft of the manuscript. KS: review and editing of the manuscript. All authors contributed to the conception and design of the study, commented on previous versions of the manuscript, read, and approved the final manuscript.

## Supplementary material

Supplementary Table 1 The normalized EMG data in APB for all participants.
